# A surface-intrinsic framework for topology-preserving hippocampal alignment and precision morphometry

**DOI:** 10.64898/2026.07.22.740127

**Published:** 2026-07-27

**Authors:** J DeKraker, D Bansal, M Snyder, BG Karat, N Talaei, M Salman, A Ngo, J Chen, E Sahlas, J Royer, DG Cabalo, MF Glasser, T Coalson, J Harwell, M Torkamani-Azar, Y Liu, J Tohka, JC Lau, AC Evans, BC Bernhardt, AR Khan

## Abstract

Accurate alignment of hippocampal anatomy across individuals remains challenging due to complex and highly variable folding patterns that are not well captured by conventional volumetric approaches. HippUnfold introduced a surface-based representation of the hippocampus, but key components—including coordinate estimation and inter-subject correspondence—were defined in the volumetric domain, making them susceptible to topological errors and interpolation artifacts. Here, we introduce a surface-intrinsic formulation of hippocampal unfolding in which geometry, intrinsic coordinates, and correspondence are defined directly on subject-specific surface manifolds. Intrinsic anterior–posterior and proximal–distal coordinates are computed by solving Laplace equations on the surface, and correspondence is established through surface-based resampling in unfolded space, replacing inverse volumetric warping. Relative to the original HippUnfold approach, this formulation improves test–retest consistency, subject identifiability, and mesh quality, while better preserving subject-specific gyral and sulcal morphology. Surface representations show reduced distortion between folded and unfolded spaces and eliminate misplaced or outlier vertices associated with volumetric warping. These improvements translate to enhanced sensitivity in a clinical application, improving lateralization of temporal lobe epilepsy. These results demonstrate that a surface-intrinsic formulation provides a principled and robust foundation for hippocampal unfolding, enabling topology-preserving alignment and more accurate characterization of inter-individual variability in health and disease.

## Full Text Availability

The license terms selected by the author(s) for this preprint version do not permit archiving in PMC. The full text is available from the preprint server.

